# The Coexistence of Hypertension and Arthritis Was Not Associated with Pain Severity in Community-Dwelling Older Adults in the United States

**DOI:** 10.3390/healthcare13050570

**Published:** 2025-03-06

**Authors:** Saud M. Alrawaili, Khalid M. Alkhathami, Mohammed G. Elsehrawy, Mohammed S. Alghamdi, Norah A. Alhwoaimel, Aqeel M. Alenazi

**Affiliations:** 1 Department of Health and Rehabilitation Sciences, Prince Sattam Bin Abdulaziz University, Al-Kharj 11942, Saudi Arabia; s.alrawaili@psau.edu.sa (S.M.A.); n.alhwoaimel@psau.edu.sa (N.A.A.); 2 Department of Health Rehabilitation, Shaqra University, Shaqra 11961, Saudi Arabia; kalkhthami@su.edu.sa; 3 Department of Nursing, Prince Sattam Bin Abdulaziz University, Al-Kharj 11942, Saudi Arabia; m.elsehrawy@psau.edu.sa; 4 Department of Medical Rehabilitation Sciences, Faculty of Applied Medical Sciences, Umm Al-Qura University, Makkah 24382, Saudi Arabia; msghamdi@uqu.edu.sa

**Keywords:** hypertensive, arthritis, pain intensity, elderly

## Abstract

**Background and Aim**: Current evidence suggests that both arthritis and hypertension (HTN) can contribute to an increase in pain severity, potentially owing to shared pathophysiological pathways. However, the extent to which these conditions jointly affect pain severity has not been well studied. The aim of this study was to explore the association between the coexistence of HTN and arthritis and their impact on pain severity among community-dwelling older adults. **Methods**: A cross-sectional design was used. Data from the Wave 2 (2010–2011) of the National Social Life, Health, and Aging Project (NSHAP) were used. Participants were community-dwelling older adults and categorized based on self-reported diagnoses into four groups: combined HTN and arthritis, HTN only, arthritis only, and neither. Pain severity was measured using the Verbal Descriptor Scale (VDS). Multiple generalized linear regression analyses were conducted with adjustments for age, sex, race, body mass index, educational level, and the use of pain and hypertension medications. **Results**: Data for 1754 participants were analyzed. The prevalence of combined HTN and arthritis was 28.4%. The prevalence of HTN only and arthritis only was 35.2% and 14.2%, respectively. Participants with both HTN and arthritis had higher pain severity compared to those with neither or only one of these conditions. After covariate adjustment, the combined HTN and arthritis group showed a significant association with higher pain severity (B = 0.39, *p* < 0.001). Similarly, the arthritis-only group also demonstrated a significant association with increased pain severity (B = 0.26, *p* = 0.002). However, the HTN alone showed no significant associations with pain severity (B = 0.014, *p* = 0.83). Compared to the arthritis-only group, combined HTN and arthritis showed a significant association with pain severity (B = 0.16, *p* = 0.049) in an unadjusted model only, and this association disappeared after adjusting for covariates (B = 0.15, *p* = 0.08). **Conclusions**: This study found no significant association between coexisting HTN and arthritis compared to arthritis alone after adjusting for covariates among community-dwelling older adults. The influence of covariates highlights the multifaceted nature of pain determinants, which emphasize the need for a multidisciplinary approach to pain management to enhance their functional capacity and overall quality of life.

## 1. Introduction

Hypertension (HTN) and arthritis are two common conditions that significantly affect the aging population worldwide and present a complex challenge in managing the health of older adults [1,2,3]. These comorbidities are prevalent among the geriatric population and significantly affect functional abilities and quality of life [4,5,6]. With an increasing aging population, it is essential to understand the coexistence of these conditions and their combined impact on pain severity in healthcare systems globally. The interplay between HTN and arthritis is particularly crucial for older adults, a group that frequently experiences the compounded effects of chronic conditions leading to symptoms and functional decline [1,7].

Arthritis, a leading cause of disability, has been extensively studied because of its direct association with pain severity. Similarly, HTN has been linked to pain modulation when it occurs concurrently with arthritis [8,9]. Chronic pain associated with arthritis can lead to mobility impairments, difficulties with activities of daily living (ADLs), and increased risk of falls among older adults [10]. Moreover, pain lasting for three or more months has been reported to be associated with a 1.6-times increased likelihood of developing frailty [10,11]. Pain in older adults is a multifaceted experience that is influenced by various physiological, psychological, and social factors [10]. Miaskowski and colleagues [10] described a conceptual model that adapts the biopsychosocial (BPS) model to understand chronic pain in older adults. This model emphasizes the complex interactions among biological, psychological, and social factors that contributes to the perception of pain experience in this population. Thus, adopting this model in understanding the impact of HTN and arthritis in pain is warranted for managing pain in older adults. Although arthritis is known to contribute to pain severity, the role of HTN as a potential exacerbating factor is still emerging in the literature [8].

The current body of literature suggests that both arthritis and HTN can contribute to an increase in pain severity, potentially owing to shared pathophysiological pathways such as inflammation, oxidative stress, and altered nociception [9,12]. However, the extent to which these conditions jointly affect pain severity has not been well studied. The few studies that have explored this relationship, such as that conducted by Alenazi et al. [8], suggest that the presence of HTN in individuals with arthritis is associated with greater pain severity, indicating a synergistic effect. This study found that 28.91% of hypertensive individuals had osteoarthritis (OA). Shi et al. [13] have reported that higher levels of HTN are associated with increased pain severity in older adults with knee OA. However, these studies often do not consider confounding variables such as the use of pain medication and hypertension medication, which could significantly influence pain outcomes [8,13]. While extensive research has independently linked HTN and arthritis to increased pain severity, there is a lack of studies examining their combined effects. Understanding the effect of these conditions on pain would be beneficial for providing preventive and therapeutic strategies for this population.

Previous research has often focused on individual conditions such as HTN and arthritis without an integrated approach that considers their coexistence and their combined impact on pain. This fragmented understanding has led to gaps in knowledge, particularly regarding how the combination of HTN and arthritis affects pain severity compared with each condition separately. Other limitations include the lack of control for other confounders that might affect the relationship between HTN and arthritis on pain severity, such as pain and hypertension medications. Thus, additional studies are needed to examine the relationship between HTN and pain severity. The primary aim of this study, therefore, was to examine the association between the coexistence of HTN and arthritis, HTN alone, or arthritis alone and their impact on pain severity among community-dwelling older adults. The secondary aim was to explore the association between the combined presence of HTN and arthritis as opposed to having arthritis alone for pain severity in this population.

## 2. Methods

### 2.1. Study Design and Participants

A cross-sectional design was used using data from Wave 2 (collected 2010–2011) of the National Social Life, Health, and Aging Project (NSHAP), a population-based study on the health and aging of community-dwelling older adults [14]. Data from Wave 2 were selected because the primary outcome variable (pain severity) was not included in Wave 1, and key confounders, such as pain medication use, were missing in Wave 3. Additionally, data in Wave 2 allowed us to minimize missing data on essential variables for the target sample, older adults aged ≥60 years. Three methods were used in collecting data: (1) individual interviews at the participants’ homes; (2) biometric measurements; and (3) self-administered questionnaires that included items about pain and health conditions such as HTN and arthritis. In Wave 2, pain-related items were assessed using the self-administered questionnaire. In the current study, we did not exclude any participants included in the database since the average Montreal Cognitive Assessment Survey Adaptation (MoCA-SA) score was 22.6, which is considered within the normal range, with 22 being the threshold for mild cognitive impairment [15].

### 2.2. Outcome Measures and Covariates

#### 2.2.1. Groups

Participants were classified into 4 groups based on their response to the following question: “Have you been diagnosed with arthritis (osteo or rheumatoid), hypertension, or both?”. These groups were as follows: (1) HTN only, (2) Arthritis only, (3) Combined HTN and Arthritis, and (4) Neither HTN nor Arthritis.

#### 2.2.2. Pain Severity

Pain severity over the previous four weeks was measured using a Verbal Descriptor Scale (VDS) for the degree of experienced pain. The scale demonstrated validity and reliability for quantifying pain severity among older adults, which considered the favored unidimensional pain scale [16,17,18]. When compared to other unidimensional pain measures, the VDS for pain demonstrated better validity and reliability, particularly for older persons with varying racial and cognitive backgrounds [19,20,21]. Using the VDS, pain intensity was assigned numerical values: no pain (0), slight pain (1), mild pain (2), moderate pain (3), severe pain (4), extreme pain (5), and most intense pain imaginable (6) [22,23].

#### 2.2.3. Covariates

Various factors known to influence pain severity in older adults were taken into account in the analyses. Demographics (age, sex, and race), education, body mass index (BMI), pain medications, and hypertension medications were included in our analyses. Age was recorded in years. The categorical variables were classified as follows: sex (male or female), race (white, black, Hispanic, and others) education (bachelor’s degree and higher, associate degree, high school, and less than high school). BMI was calculated using weight in kilograms and height in meters. Pain medications included eight types (i.e., salicylates, COX-2 inhibitors, analgesic combinations, non-steroidal anti-inflammatory, narcotic analgesics, miscellaneous analgesics, general analgesics, and central nervous system drugs). Participants were asked to report on their use of pain medication type with a yes or no response. The responses were subsequently combined into a single variable, “pain medications”, representing yes/no for the use of any of the eight medication types. Four types of hypertension medications were included: non-cardio selective beta blockers, cardio selective beta blockers, angiotensin II inhibitors, and antihypertensive combinations. Participants were asked to indicate their use of each type of hypertension medication with a yes or no response. The responses were then combined into a single variable, “hypertension medications”, representing yes/no for the use of any of the four medication types.

### 2.3. Statistical Analysis

Descriptive statistics were computed for demographics and clinical characteristics for all participants. Group comparisons for demographic and clinical variables were conducted using one-way ANOVA for continuous measures and Chi-square tests for categorical data. Further, a post hoc analysis was conducted to compare the differences between groups in pain severity using Tukey’s Least Significant Difference (LSD).

To examine the association between groups with pain severity, multiple generalized linear regression analyses were conducted. Two models were developed: model 1 was unadjusted, while model 2 was adjusted for age, sex, race, educational level, BMI, and the use of pain and hypertension medications. The results were reported as unstandardized coefficients (B) with standard errors (SEs).

Further analysis was conducted focusing exclusively on individuals with arthritis, with and without HTN. Multiple generalized linear regression analyses were utilized to examine the association between groups including the combined HTN and arthritis group compared to the arthritis-only group. Model 1 was an unadjusted model, while model 2 was adjusted for age, sex, race, educational level, BMI, pain medications, and hypertension medications. The results were presented as unstandardized coefficients (B) with standard error.

Sensitivity analysis was conducted based on pain medications (8 types) and hypertension medications (4 types) for model 2. When the results remained similar after sensitivity analysis, the results of model 2 were reported based on the original analyses, combining any pain medications in one variable and combining any hypertension medications in one variable. The alpha level was set at 0.05 for all the analyses, and an IBM SPSS for Mac version 25.0 (SPSS Inc. Chicago, IL, USA) was used.

## 3. Results

A total of 3196 community-dwelling older adults were initially included in the study. The total number included in the final analysis, however, was 1754, due to missing data on key variables such as the severity of pain and self-reported arthritis and/or HTN. The prevalence of coexisting HTN and arthritis was 28.4% among older adults in the current study. The prevalence of HTN only and arthritis only was 35.2% and 14.2%, respectively. Participants included in this wave of the study were aged 62 to 91 years of age. There were significant differences between groups in age, sex, BMI, race, and educational level. The mean age was highest among those with coexisting HTN and arthritis (74 years) and lowest in the neither group. Female percentages were significantly higher in the combined HTN and arthritis group (62.5%) and the arthritis-only group (64.3%) compared to the HTN-only and neither condition groups. BMI was the highest among those in the combined HTN and arthritis group (31.49 kg/m^2^) and lowest among those with neither condition (28.2 kg/m^2^). Additionally, the usage of pain and hypertension medications significantly (*p* < 0.001) differed among the groups, with the highest use reported in the combined HTN and arthritis group (74.0%) and arthritis-only group (70.8%). Participants with both HTN and arthritis reported the highest pain severity (mean = 3.22), whereas those without either condition experienced the lowest pain severity (mean = 2.72). Post hoc analysis using LSD showed significant differences across groups in pain severity. The combined HTN and arthritis group had a significant increased pain severity when compared to other groups including the neither group (mean difference = 0.49, *p* < 0.001), arthritis-only group (mean difference = 0.16, *p* = 0.049), and HTN-only group (mean difference = 0.44, *p* < 0.001). Table 1 shows demographic and clinical variables across groups.

Table 2 presents the results of the multiple generalized linear models examining the association between groups and pain severity. In model 1, which was the unadjusted model, significant associations with pain severity were found for two groups: those with combined HTN and arthritis (B = 0.49, *p* < 0.001) and those with arthritis only (B = 0.34, *p* = 0.001). Model 2, which was adjusted for age, sex, BMI, race, education, the use of pain and hypertension medications showed similar results. Specifically, the combined HTN and arthritis group showed a significant association with pain severity (B = 0.39, *p* < 0.001), as did the arthritis-only group (B = 0.26, *p* = 0.002). However, HTN alone showed no significant associations with pain severity (B = 0.014, *p* = 0.83). The adjusted R^2^ was 0.019 and 0.057 for model 1 and model 2, respectively.

Table 3 presents the results of multiple generalized linear models assessing the association between arthritis (with and without HTN) and pain severity. In the unadjusted model (model 1), the combined HTN and arthritis group showed significant association with increased pain severity (B = 0.16, *p* = 0.049). However, after adjusting for covariates in model 2, this association was no longer statistically significant (B = 0.15, *p* = 0.08). The adjusted R^2^ was 0.005 and 0.061 for model 1 and model 2, respectively.

The results of the sensitivity analysis showed similar results based on including each pain medication (eight classes) and each hypertension medication (four classes). The combined HTN and arthritis group remained significantly associated with increased pain severity (B = 33, *p* < 0.001), and the arthritis-only group remained significantly associated with increased pain severity (B = 0.24, *p* = 0.015) in the sensitivity analysis. Therefore, the original models were reported in the tables without sensitivity analysis.

## 4. Discussion

The primary objective of the study was to explore the relationship between the presence of hypertension (HTN) and arthritis, individually or in combination, and their impact on pain severity among community-dwelling older adults. In this study, we found a notable prevalence of combined HTN and arthritis in community-dwelling older adults, with 22.6% having both conditions. Additionally, 11.2% had HTN only and 37.9% had arthritis only, indicating a considerable coexistence of these conditions and their potential impact on pain severity in the geriatric population. In alignment with these findings, Alenazi et al. [8] reported that 28.91% of individuals with arthritis also had HTN, noting a significant association between HTN and increased joint pain severity in people with arthritis. Additionally, Li et al. [9] found that in community-dwelling older adults with hypertension, 57.12% had bothersome pain and 56.21% had pain that limited their activities. The authors also found that arthritis was one of the comorbidities that significantly increased the likelihood of experiencing bothersome pain in older adults [9]. In contrast, the study by Bae et al. [24] found an inverse relationship between HTN and the prevalence of low back pain and arthritis, suggesting that hypertension might be associated with lower pain perception in these conditions. These studies collectively highlight the complex and varied nature of the relationship between HTN, arthritis, and pain. Varied conclusions across studies can be attributed to differences in participant demographic characteristics, as well as differences in study methodologies.

Our findings demonstrate a significant association between the coexistence of HTN and arthritis, as well as arthritis alone, with an increase in pain severity among community-dwelling older adults. These associations remain consistent after adjusting for demographic factors and medication usage. However, when using a subgroup analysis comparing coexisting HTN and arthritis to arthritis only, the significant association with pain severity disappeared after adjustments to covariates including pain and HTN medications. This observation suggests the potential influence of mediating covariate variables included in the analysis. Furthermore, the modest R^2^ values from the regression models indicate that they explain only a small proportion of the variance in pain severity. These results highlight the multifaceted nature of pain determinants in older adults and emphasize the need for a further exploration of additional contributing factors. Our finding was inconsistent with the results of recent studies explored the association between HTN and pain severity among the arthritis population [8,13]. In one study, the authors found that patients with stage 2 HTN reported more severe knee pain severity than those with the normal blood pressure group, elevated HTN group, and stage 1 HTN group [13]. Additionally, a study found that HTN was significantly associated with increased joint pain severity among individuals with OA regardless of location [8]. The previous studies used more sensitive pain scales that were specific to knees, while our study used VDS for pain severity that may limit the generalizability of our results.

In our study, a small but significant difference was found in pain severity between older adults who have both HTN and arthritis and those who have neither. The group with both conditions had a slightly higher average pain score (3.22) compared to those without either condition (2.74). Although the difference in pain scores is modest, it highlights the importance of managing these conditions together in older adults as their coexistence is prevalent. Regardless of the pain measures used, previous research studies have found an increasing trend in pain severity in older adults who have HTN, arthritis or both [8,13]. These findings emphasize the importance of regular pain assessment especially for those with hypertension and arthritis.

Metabolic syndrome, including conditions such as HTN, shares several pathophysiological mechanisms with arthritis (osteoarthritis in particular, OA), such as inflammatory aging, oxidative stress, and dysfunction in vascular endothelial cells. These mechanisms contribute to cartilage damage and an increase in OA symptoms, notably pain [9]. The mechanism of the increased pain severity and its association with HTN has been examined in several studies [9,25,26,27]. One proposed mechanism is that HTN could lead to subchondral bone ischemia, adversely affecting nutrient delivery to cartilage and hindering bone remodeling processes [25,27]. Additionally, damage to vascular endothelial cells caused by HTN might prompt the secretion of prostaglandins, potentially leading to increased joint inflammation and subsequent cartilage damage [26].

Along with existing research, our findings have important implications for health care professionals concerned with the management of community-dwelling older adults. Since previous research has linked coexisting HTN and arthritis to a decline in physical performance measures such as gait speed and functionality [7], in addition to severity of pain, it is recommended that health care providers screen for HTN, particularly in older adults with arthritis. This is due to the substantial association between HTN and increased pain severity in this population. When evaluating pain severity in older adults, it is important to use tools that are easily understood and can be effectively communicated. Comprehensive pain management should include a multidisciplinary approach to create tailored interventions (e.g., pharmacologic and/or non-pharmacologic options) to enhance their functional ability and overall quality of life.

Research employing secondary data analysis inherently carries limitations. The cross-sectional design limits the causality inference from the current study, and the findings are limited to a possible association. The diagnoses of HTN and arthritis were determined through self-reported data, with participants providing simple yes/no answers. This approach could impact the accuracy of the group membership used in this study. For the arthritis question, participants were only asked to confirm whether they had any type of arthritis, without any further specificity. This lack of detail regarding the exact type of arthritis (osteoarthritis or rheumatoid) may affect the study’s generalizability, although the majority of arthritis is osteoarthritis among older adults. The distinct clinical and pathophysiological profiles of both types and their treatment approaches may also influence the associations observed. Moreover, the assessment of pain was limited as it was reported as generalized pain severity without reference to a specific affected site or joint or location and was limited to one measure (i.e., VDS). Future research should examine pain in a multidimensional approach. This may lead to an underestimation of the relationship between specific joint pain and the conditions studied. Although pain was not specified as being acute or chronic in the current study, it is not likely to be acute due to the question about pain in the past 4 weeks. The lack of biomarkers related to HTN and arthritis is another limitation that should be acknowledged in the current study. Linking biomarkers to pain severity should be addressed in future studies. Further work is needed to explore the relationship between HTN and arthritis with joint pain locations, especially for lower extremity. Future research is needed to explore the determinants of pain severity in older adults given the complexity of the aging process and the accompanying health conditions. Furthermore, investigating the effect of different pain and hypertension medications on the pain severity in this population is warranted.

## 5. Conclusions

This study revealed no significant association between coexisting HTN and arthritis compared to arthritis alone after adjusting for covariates among community-dwelling older adults. Although an initial association between coexisting HTN and arthritis was observed in the unadjusted model, it was no longer significant in the adjusted model. The influence of covariates, particularly pain and HTN medications, suggests that these factors may partially mediate the observed relationship, highlighting the multifactorial nature of pain determinants in older adults. The prevalence of these conditions emphasizes the importance of a multidisciplinary approach to pain management in older adults to enhance their functional capacity and overall quality of life.

## Figures and Tables

**Table 1:** Participants’ demographic and clinical characteristics.

Variables	Combined HTN and Arthritis n = 499	Arthritis Only n = 249	HTN Only n = 617	Neither HTN Nor Arthritis n = 389	*p*-Value
Age, years (mean ± SD)	74 ± 7	74 ± 7	72 ± 7	71 ± 7	<0.001
Sex, female, n (%) within groups	312 (62.5)	160 (64.3)	298 (48.3)	183 (47.0)	<0.001
BMI, kg/m^2^ (mean ± SD)	31.51 ± 6.9	28.50 ± 5.7	29.95 ± 6.1	28.15 ± 6.2	<0.001
Race, n (%) within groups					<0.001
White	345 (69.3)	196 (79.4)	450 (73.2)	329 (84.6)	
Black	91 (18.3)	21 (8.5)	81 (13.2)	27 (6.9)	
Hispanic	53 (10.6)	22 (8.9)	61 (9.9)	29 (7.5)	
Others	9 (1.8)	8 (3.2)	23 (3.7)	4 (1.0)	
Education, n (%) within groups					<0.001
Less than high school	123 (24.6)	41 (16.5)	100 (16.2)	52 (13.4)	
High school	138 (27.7)	56 (22.5)	158 (25.6)	87 (22.4)	
Some college/associate degree	143 (28.7)	81 (32.5)	211 (34.2)	122 (31.4)	
Bachelors or more	95 (19.0)	71 (28.5)	148 (24.0)	128 (32.9)	
Hypertension medications, yes, n (%) within groups	300 (61.0)	50 (20.6)	381 (62.4)	75 (19.6)	<0.001
Pain medications, yes, n (%) within groups	364 (74.0)	172 (70.8)	371 (60.7)	201 (52.5)	<0.001
Pain severity (mean ± SD)	3.22 ± 1	3.06 ± 1	2.78 ± 1	2.72 ± 1	<0.001

Note: SD: standard deviation, BMI: body mass index.

**Table 2:** Linear regression analyses for the association between groups and pain severity.

Groups	Model 1 (n = 1754)			Model 2 (n = 1637)		
	B	SE	*p*-Value	B	SE	*p*-Value
Combined HTN and Arthritis	0.49	0.06	<0.0001	0.39	0.07	<0.0001
Arthritis only	0.34	0.08	<0.001	0.26	0.08	0.002
HTN only	0.06	0.06	0.37	0.014	0.07	0.83
Neither HTN nor Arthritis	reference			reference		
R^2^	0.019			0.057		

Note: n = number of patients; SE = standard error; HTN = hypertension; model 1 = unadjusted model; model 2 = adjusted for age, sex, race, BMI, education, pain medications, and hypertension medications.

**Table 3:** Linear regression analyses for the association between combined HTN and arthritis compared to arthritis only with pain severity.

Groups	Model 1 (n = 748)			Model 2 (n = 691)		
	B	SE	*p*-Value	B	SE	*p*-Value
Combined HTN and arthritis	0.16	0.08	0.049	0.15	0.09	0.08
Arthritis only	Reference			Reference		
R^2^	0.005			0.061		

Note: n = number of patients; SE = standard error; HTN = hypertension; model 1 = unadjusted model; model 2 = adjusted for age, sex, race, BMI, education, pain medications, and hypertension medications.

## Data Availability

The NSHAP data are publicly available through the National Archive of Computerized Data on Aging (NACDA, https://www.icpsr.umich.edu/icpsrweb/NACDA/ (accessed on 19 November 2023)).
